# Differences in COVID-19 testing and adverse outcomes by race, ethnicity, sex, and health system setting in a large diverse US cohort

**DOI:** 10.1371/journal.pone.0276742

**Published:** 2022-11-23

**Authors:** Celeena Jefferson, Eric Watson, Julia M. Certa, Kirsha S. Gordon, Lesley S. Park, Gypsyamber D’Souza, Lorie Benning, Alison G. Abraham, Deana Agil, Sonia Napravnik, Michael J. Silverberg, Wendy A. Leyden, Jacek Skarbinski, Carolyn Williams, Keri N. Althoff, Michael A. Horberg, on behalf of the NA-ACCORD Corona-Infectious-Virus Epidemiology Team (CIVET)

**Affiliations:** 1 Kaiser Permanente Mid-Atlantic Permanente Medical Group, Mid-Atlantic Permanente Research Institute, Rockville, Maryland, United States of America; 2 United Health Group, Fredrick, Maryland, United States of America; 3 Yale School of Medicine, Department of General Internal Medicine, New Haven, Connecticut, United States of America; 4 VA Connecticut Healthcare System, West Haven, Connecticut, United States of America; 5 Stanford Center for Population Health Sciences, Stanford University School of Medicine, Palo Alto, California, United States of America; 6 Department of Epidemiology, Johns Hopkins Bloomberg School of Public Health, Baltimore, Maryland, United States of America; 7 Department of Epidemiology, Anschutz Medical Campus, University of Colorado, Aurora, Colorado, United States of America; 8 Department of Medicine, University of North Carolina at Chapel Hill, Chapel Hill, North Carolina, United States of America; 9 Division of Research, Kaiser Permanente Northern California, Oakland, California, United States of America; 10 Division of AIDS, National Institute of Allergy and Infectious Diseases, Rockville, Maryland, United States of America; LSU Health Sciences Center New Orleans: Louisiana State University Health Sciences Center, UNITED STATES

## Abstract

**Background:**

Racial/ethnic disparities during the first six months of the COVID-19 pandemic led to differences in COVID-19 testing and adverse outcomes. We examine differences in testing and adverse outcomes by race/ethnicity and sex across a geographically diverse and system-based COVID-19 cohort collaboration.

**Methods:**

Observational study among adults (≥18 years) within six US cohorts from March 1, 2020 to August 31, 2020 using data from electronic health record and patient reporting. Race/ethnicity and sex as risk factors were primary exposures, with health system type (integrated health system, academic health system, or interval cohort) as secondary. Proportions measured SARS-CoV-2 testing and positivity; attributed hospitalization and death related to COVID-19. Relative risk ratios (RR) with 95% confidence intervals quantified associations between exposures and main outcomes.

**Results:**

5,958,908 patients were included. Hispanic patients had the highest proportions of SARS-CoV-2 testing (16%) and positivity (18%), while Asian/Pacific Islander patients had the lowest portions tested (11%) and White patients had the lowest positivity rates (5%). Men had a lower likelihood of testing (RR = 0.90 [0.89–0.90]) and a higher positivity risk (RR = 1.16 [1.14–1.18]) compared to women. Black patients were more likely to have COVID-19-related hospitalizations (RR = 1.36 [1.28–1.44]) and death (RR = 1.17 [1.03–1.32]) compared with White patients. Men were more likely to be hospitalized (RR = 1.30 [1.16–1.22]) or die (RR = 1.70 [1.53–1.89]) compared to women. These racial/ethnic and sex differences were reflected in both health system types.

**Conclusions:**

This study supports evidence of disparities by race/ethnicity and sex during the COVID-19 pandemic that persisted even in healthcare settings with reduced barriers to accessing care. Further research is needed to understand and prevent the drivers that resulted in higher burdens of morbidity among certain Black patients and men.

## Introduction

In the United States, medical literature has exposed significant disparities in testing, infection, and adverse outcomes related to COVID-19 (caused by SARS-CoV-2); in particular, Black and Hispanic Americans have been disproportionately impacted by COVID-19 [1–19]. However, few large-scale multi-regional investigations into these disparities have been reported [1, 19, 20], none of which describe differences by type of health system.

Early data suggested COVID-19 testing and positivity differed by sex and race/ethnicity, with reduced testing among minority populations but higher positivity among Black and Hispanic Americans compared to White Americans [9, 13, 21, 22] as well as adverse COVID-19 outcomes, such as hospitalizations and deaths [1, 12]. These disparities in access to COVID-19 diagnosis and care are associated with long-standing inequities in the US [23]. The impact of differential exposure to SARS-CoV-2, inequities in health care access, and underlying comorbid conditions remain unresolved.

We sought to evaluate racial/ethnic and sex differences in SARS-CoV-2 testing and positivity and COVID-19-related hospitalization and death within six demographically and geographically diverse cohorts, and to determine if these differences persist in the context of different health system models.

## Methods

### CIVET cohort

The Corona-Infectious-Virus Epidemiology Team (CIVET) is a collaboration of three integrated health systems, two academic health centers, and one interval cohort which was a clinical cohort of people with and without HIV. [24] that participate in the North American AIDS Cohort Collaboration on Research and Design (NA-ACCORD): [25] Kaiser Permanente Northern California (KPNC); Kaiser Permanente Mid-Atlantic States (KPMAS); MACS/WIHS Combined Cohort Study (MWCCS); [24] University of North Carolina at Chapel Hill HIV Clinical Cohort (UCHCC); [26] Vanderbilt Comprehensive Care Clinic HIV Cohort (VBCCC); and Veterans Aging Cohort Study (VACS) [27]. In March 2020, these sites had either initiated COVID-19 clinical cohorts within their individual sites, linking existing large-scale cohorts of people to electronic COVID-19 testing, symptoms, and diagnosis data, or surveys related to COVID-19.

The CIVET cohort included individuals from each site who met study inclusion and exclusion criteria. Each cohort restricted their study population to include: 1) individuals who were alive as of March 1, 2020; and 2) individuals who were “in care” or “in cohort,” which was operationalized differently for each cohort but based on individuals who had recently interacted with the health system or interval study (S1 Table).

### Study design

We conducted a retrospective analysis of the CIVET cohort to investigate differences by race/ethnicity and sex in COVID-19-related outcomes (testing, positivity, hospitalization, and death) among adults (≥18 years old) between March 1, 2020 and August 31, 2020. The study period was restricted to the initial viral serotype timeframe to limit possible differences by variants. An index date for each participant was defined as either: 1) the first positive SARS-CoV-2 polymerase chain reaction (PCR) test laboratory date, 2) the last negative or pending/invalid SARS-CoV-2 PCR test laboratory date (if no positive test found), or 3) March 1, 2020 (for those not found to have been PCR tested). Patient-level data were aggregated at each site and sent to KPMAS. American Community Survey (ACS) data from the US Census Bureau was also collected for population level comparisons [28, 29].

### Exposure

Sex (male, female, other, unknown) was identified by data availability of sex at birth or self-reported sex/gender. Other and unknown sex categories were too small and excluded. Race/ethnicity was self-reported and classified as non-Hispanic White, non-Hispanic Black, Hispanic, Asian/Pacific Islander (API), other, and unknown (excluded from analyses). To determine the robustness of racial/ethnic and sex differences to barriers to healthcare, participating clinical cohorts were further grouped by type of health system, defined as integrated health systems (KPNC, KPMAS, VACS), academic health systems (UCHCC, VBCCC), and interval cohort (MWCCS). Integrated systems represented lower barriers to care, with membership allowing patients to readily communicate with healthcare professionals, initiate e-visits, and access SARS-CoV-2 testing. In contrast, academic systems are open to the public and often serve as safety-net providers in their communities, but acknowledging these differences are fluid [30]. The interval cohort is an HIV cohort consisting of persons with and without HIV.

### Main outcomes

#### SARS-CoV-2 testing and positivity

SARS-CoV-2 RT-PCR testing status (yes/no), date, and result (positive, negative, pending/invalid) were extracted from electronic health record (EHR) systems and reported as composite data from each cohort. During the study period (March–August 2020), routine testing was limited and generally at the discretion of a clinician due to changes in testing availability and guidelines, which differed by state and healthcare system. Testing was calculated as those tested among the entire study population. Positivity was calculated as the number testing positive among those tested.

#### Hospitalization

Patients hospitalized with clinical syndrome of COVID-19 within seven days prior to and 45 days after their first positive SARS-CoV-2 test were included. To identify inpatient encounters associated with clinical COVID-19, the following International Classification of Disease, Tenth Edition (ICD-10) codes were pulled from hospitalization records: B97.2x, J12.8x, J12.9, J18.x, J20.8, J22, J80, J96.0x, J96.2x, J96.9x, M35.8x, A41.x, R65.x. We were unable to discriminate between COVID-19 as the primary cause of hospitalization versus hospitalization primarily for another cause but with COVID-19, so patients with non-specific COVID-19 codes were included [31]. Additionally, as the pandemic progressed, more specific ICD codes were activated [31–34].

#### Death

Mortality data and the death outcome were measured by patients who were known to be deceased after their first positive SARS-CoV-2 test during the study period. The rest were presumed alive. Death data was obtained from EHR systems, claims, the VA Vital Status File, which includes data from inpatient records, the VA Beneficiary Identification Records Locator Subsystem, Social Security Administration, and the Center for Medicare and Medicaid Services.

### Statistical analysis

Each site shared standardized de-identified, aggregate tables with KPMAS, and were pooled for analyses. We calculated the proportion tested (among the study population), the proportion with detectable SARS-CoV-2 PCR test results (among those tested), and the proportions who were hospitalized and died (among those with detectable SARS-CoV-2 PCR results). We used an *a priori* study team consensus-driven minimal important difference of +/-3% to guide our interpretation of differences in the outcomes by race/ethnicity and sex, and a chi-squared (χ^2^) test statistic (α = 0.05) in the pooled aggregated data for interpretation of statistical significance.

We calculated Cochran–Mantel–Haenszel stratification-adjusted relative risk ratios (RR) and associated 95% confidence intervals (CI) to quantify associations between race/ethnicity and sex and study outcomes. We adjusted for race/ethnicity or sex to isolate risk factor effects and stratified the estimated outcome proportions and the RRs by clinical cohort health system type (integrated health system, academic health system, or interval cohort) to determine if the differences persisted in the setting of lowered barriers to healthcare. Due to differences in the size and cohort selection criteria (VACS includes people without HIV who are demographically matched to people with HIV), the analyses in the integrated health systems group were further stratified by cohort (KPNC, KPMAS, and VACS) to determine the robustness of the intragroup differences; it was determined that the three cohorts were similar enough to remain cohesively grouped. Analyses were performed using SAS 9.4 (Cary, NC). Approval was obtained from each participating site’s institutional review board, which included waivers of written informed consent.

## Results

A total of 5,958,908 patients (5,955,269 from clinical cohorts and 3,639 from the interval cohort) were included in the study population, of whom 5,724 were hospitalized with clinical syndrome COVID-19 diagnoses and 1,399 died after a detectable SARS-CoV-2 PCR test result (Table 1). The overall study population was 45% White, 13% Black, 15% Hispanic, 14% Asian/Pacific Islander, 53% female, and 71% <60 years old. Comparatively, the US population, based on 2020 American Community Survey (ACS) estimates, was 60% White, 12% Black, 18% Hispanic, 6% Asian/Pacific Islander, 51% female, and 71% <60 years old [28, 29]. Fifty-seven percent (57%) were commercially insured, 32% with publicly supported insurance (Medicare, Medicaid, ACA), 25% received care in an academic health system, 75% in an integrated health system, and <1% were enrolled in the single interval cohort. Most notable distinctions in cohort distributions include primarily older, >60 years old, in cohorts E (57%) and S (40%); higher proportion of White in cohorts V (63%) and T(70%); higher proportion of black in cohorts C (35%), E (46%) and S (41%); higher proportion of Hispanic in cohorts C (12%) and I (20%); large majority of males in cohort E (96%); and high proportion of Medicare in cohort V (31%) (Table 2).

**Table 1:** Patient characteristics by SARS-CoV-2 testing status, March 1, 2020-August 31, 2020.

Characteristic	Overall	Testing Status		
		Positive	Negative	Not Tested
**Total Patients (N, %):**	**5,958,908**	**66,014 (1%)**	**661,058 (11%)**	**5,231,771 (88%)**
**Health System Type (N, %)**				
Academic Health Systems	1,473,301 (25%)	15,249 (23%)	138,789 (21%)	1,319,263 (25%)
Integrated Health Systems	4,481,968 (75%)	50,661 (77%)	521,038 (79%)	3,910,206 (75%)
Interval HIV Cohort	3,639 (<1%)	104 (<1%)	1,231 (<1%)	2,302 (<1%)
**Age (N, %):**				
18–29	1,143,859 (19%)	15,955 (24%)	116,990 (18%)	1,010,910 (19%)
30–39	1,070,408 (18%)	13,283 (20%)	124,196 (19%)	932,922 (18%)
40–49	968,795 (16%)	12,643 (19%)	106,737 (16%)	849,407 (16%)
50–59	1,024,366 (17%)	11,778 (18%)	115,444 (17%)	897,134 (17%)
60–69	917,935 (15%)	7,453 (11%)	103,238 (16%)	807,216 (15%)
70+	833,545 (14%)	4,902 (7%)	94,453 (14%)	734,182 (14%)
**Race/Ethnicity (N, %):**				
White	2,705,253 (45%)	16,413 (25%)	312,053 (47%)	2,376,772 (45%)
Black	756,543 (13%)	11,542 (17%)	92,188 (14%)	652,772 (12%)
Hispanic	874,131 (15%)	24,576 (37%)	113,118 (17%)	736,432 (14%)
Asian/Pacific Islander	811,271 (14%)	5,813 (9%)	81,972 (12%)	723,485 (14%)
Other	148,822 (2%)	1,421 (2%)	19,006 (3%)	128,394 (2%)
Unknown	662,888 (11%)	6,249 (9%)	42,721 (6%)	613,916 (12%)
**Sex (N, %):**				
Male	2,802,087 (47%)	31,453 (48%)	286,358 (43%)	2,484,223 (47%)
Female	3,155,582 (53%)	34,552 (52%)	374,588 (57%)	2,746,430 (52%)
Unknown	1,233 (<1%)	9 (<1%)	112 (<1%)	1,112 (<1%)
Other	6 (<1%)	0 (0%)	0 (0%)	6 (<1%)
**Insurance Status/Line of Business (N, %):**				
Commercial	3,418,447 (57%)	40,425 (61%)	381,110 (58%)	2,996,906 (57%)
Medicare	1,195,814 (20%)	7,211 (11%)	143,052 (22%)	1,045,545 (20%)
Medicaid	318,080 (5%)	4,690 (7%)	38,722 (6%)	274,663 (5%)
ACA Exchange	390,387 (7%)	4,117 (6%)	35,531 (5%)	350,738 (7%)
Charity Care	2,463 (0%)	42 (0%)	195 (0%)	2,226 (0%)
Other LOB	86,961 (1%)	1,062 (2%)	5,910 (1%)	79,989 (2%)
No Insurance	155,062 (3%)	2,477 (4%)	15,098 (2%)	137,487 (3%)
Unknown	391,694 (7%)	5,990 (9%)	41,440 (6%)	344,217 (7%)
**Hospitalized with Clinical Syndrome COVID-19 Diagnoses (N, %)**	24,451 (<1%)	5,724 (9%)	18,722 (3%)	
**Known Dead (N, %)**	1,399 (<1%)	1,399 (2%)		

Abbreviations: ACA, Affordable Care Act; LOB, line of business

Cell percentages are calculated from SARS-CoV-2 Testing Status (column) totals.

**Table 2:** Patient characteristics by Cohort, March 1, 2020-August 31, 2020.

Characteristic	Overall	Cohorts					
		C	I	V	E	T	S
**Total Patients (N, %):**	**5,958,908**	686,438 (12%)	3,687,471 (62%)	851,184 (14%)	108,059 (2%)	622,117 (10%)	3,639 (0%)
**Age (N, %):**							
18–29	1,143,859 (19%)	145,116 (21%)	745,778 (20%)	129,265 (15%)	867 (1%)	122,809 (20%)	24 (1%)
30–39	1,070,408 (18%)	128,208 (19%)	720,534 (20%)	113,520 (13%)	7,379 (7%)	100,473 (16%)	294 (8%)
40–49	968,795 (16%)	113,793 (17%)	626,935 (17%)	123,640 (15%)	9,926 (9%)	93,850 (15%)	651 (18%)
50–59	1,024,366 (17%)	121,316 (18%)	622,358 (17%)	148,750 (17%)	27,805 (26%)	102,923 (17%)	1,214 (33%)
60–69	917,935 (15%)	101,288 (15%)	521,600 (14%)	153,047 (18%)	38,979 (36%)	101,964 (16%)	1,057 (29%)
70+	833,545 (14%)	76,717 (11%)	450,266 (12%)	182,962 (21%)	23,103 (21%)	100,098 (16%)	399 (11%)
**Race/Ethnicity (N, %):**							
White	2,705,253 (45%)	181,078 (26%)	1,515,317 (41%)	536,741 (63%)	37,868 (35%)	432,966 (70%)	1,283 (35%)
Black	756,543 (13%)	240,235 (35%)	233,158 (6%)	170,748 (20%)	49,246 (46%)	61,649 (10%)	1,507 (41%)
Hispanic	874,131 (15%)	79,862 (12%)	729,662 (20%)	49,299 (6%)	9,111 (8%)	6,017 (1%)	180 (5%)
Asian/Pacific Islander	811,271 (14%)	82,311 (12%)	702,442 (19%)	14,021 (2%)	1,128 (1%)	11,342 (2%)	27 (1%)
Other	148,822 (2%)	15,358 (2%)	106,462 (3%)	18,283 (2%)	1,479 (1%)	6,608 (1%)	632 (17%)
Unknown	662,888 (11%)	87,594 (13%)	400,430 (11%)	62,092 (7%)	9,227 (9%)	103,535 (17%)	10 (0%)
**Sex (N, %):**							
Male	2,802,087 (47%)	315,758 (46%)	1,782,014 (48%)	347,972 (41%)	104,113 (96%)	250,514 (40%)	1,716 (47%)
Female	3,155,582 (53%)	370,680 (54%)	1,904,839 (52%)	503,024 (59%)	3,946 (4%)	371,170 (60%)	1,923 (53%)
Unknown	1,233 (0%)	0 (0%)	612 (0%)	188 (0%)	0 (0%)	433 (0%)	0 (0%)
Other	6 (0%)	0 (0%)	6 (0%)	0 (0%)	0 (0%)	0 (0%)	0 (0%)
**Insurance Status/Line of Business (N, %):**							
Commercial	3,418,447 (57%)	414,634 (60%)	2,429,919 (66%)	384,073 (45%)	0 (0%)	189,821 (31%)	0 (0%)
Medicare	1,195,814 (20%)	114,124 (17%)	705,581 (19%)	265,352 (31%)	0 (0%)	110,757 (18%)	0 (0%)
Medicaid	318,080 (5%)	59,140 (9%)	172,579 (5%)	61,821 (7%)	0 (0%)	24,540 (4%)	0 (0%)
ACA	390,387 (7%)	41,723 (6%)	338,869 (9%)	0 (0%)	0 (0%)	9,795 (2%)	0 (0%)
Charity Care	2,463 (0%)	857 (0%)	1,606 (0%)	0 (0%)	0 (0%)	0 (0%)	0 (0%)
Other LOB	86,961 (1%)	4,167 (1%)	28,198 (1%)	36,669 (4%)	0 (0%)	17,927 (3%)	0 (0%)
No Insurance	155,062 (3%)	51,793 (8%)	0 (0%)	103,269 (12%)	0 (0%)	0 (0%)	0 (0%)
Unknown	391,694 (7%)	0 (0%)	10,719 (0%)	0 (0%)	108,059 (100%)	269,277 (43%)	3,639 (100%)
**Hospitalized for any reason (N, %)**	83,833 (1%)	6,901 (1%)	31,868 (1%)	14,775 (2%)	4,584 (4%)	25,705 (4%)	0 (0%)
**Hospitalized with Clinical Syndrome COVID-19 Diagnoses (N, %)**	24,451 (0%)	1,209 (0%)	13,898 (0%)	5,143 (1%)	864 (1%)	3,316 (1%)	21 (1%)
**Known Dead (N, %)**	1,399 (0%)	283 (0%)	578 (0%)	324 (0%)	134 (0%)	80 (0%)	0 (0%)

Specific CIVET cohort site names have been masked.

During the study period, 1% of the study population tested positive for SARS-CoV-2, 11% tested negative, and 88% had no evidence of testing. Demographics differed by testing status. Hispanic patients formed the plurality of those testing positive (37%) but only 14% of those not tested, while White patients formed the plurality of those testing negative (47%), aligning with the race distribution in those who were not tested. There was a greater proportion of women among those who tested positive (52%). The age distribution among those with positive test results was skewed to younger ages, with 82% <60 years old, whereas the age distribution among those who tested negative was more evenly distributed across age groups

Those hospitalized with a clinical COVID-19 diagnosis (5,580) were predominantly Hispanic (1,979; 35%) and male (3,224; 58%). Those who died after a detectable SARS-CoV-2 test result (1,371) were more likely to be White (511; 37%) and male (820; 60%).

### Racial/ethnic and sex differences in COVID-19 outcomes

**Fig 1:**
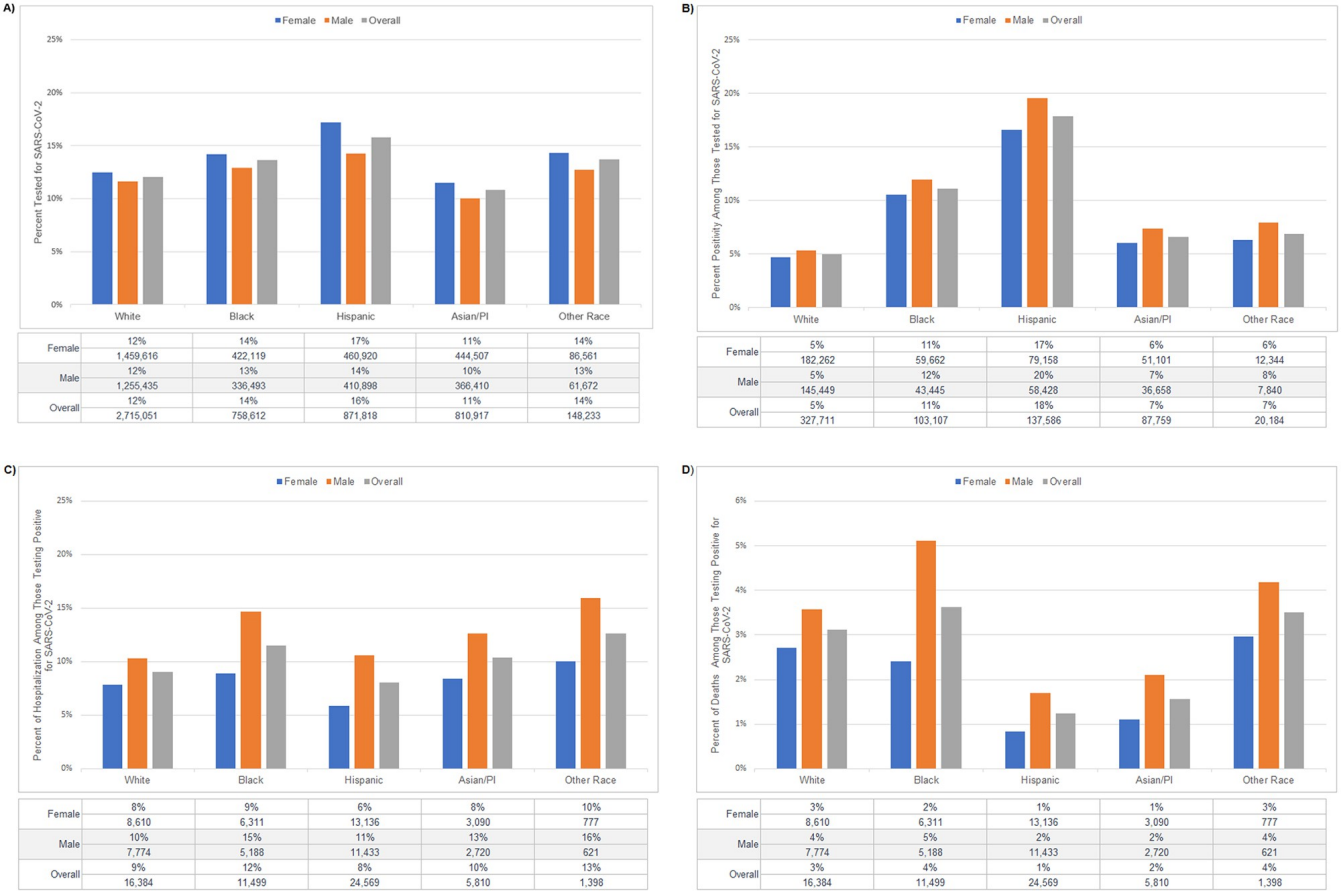
Racial/ethnic and sex differences in COVID-19 outcomes in clinical cohorts for (A) SARS-CoV-2 testing, (B) positivity, (C) hospitalization, and (D) death. Overall percentages are calculated among clinical cohort population (N = 5,304,631). Percentages by sex are calculated out of the overall number of members within each race group. Asian/PI = Asian/Pacific Islander.

Among the six clinical cohorts, Hispanic patients had the highest proportion tested for SARS-CoV-2, while Asian/Pacific Islander had the lowest proportions tested, overall and when stratified by sex (Fig 1A). After controlling for sex, these differences persisted (Hispanic RR = 1.31 [95% CI: 1.30–1.32]; API RR = 0.90 [0.89–0.90]; Table 3a). After controlling for race/ethnicity to investigate sex differences, men had lower proportions of testing than women (RR = 0.90 [0.89–0.90]).

**Table 3:** Risk ratios of (A) SARS-CoV-2 testing, (B) positivity, (C) hospitalization, and (D) death. Risk ratios by race compared to White, Non-Hispanic persons; risk ratios by sex compared to Female persons. Specific CIVET cohort site names have been masked.

**a. Testing risk ratios & 95% confidence intervals**					
**CIVET Cohort**	**RACE** *				**SEX** **
	API	BLA	HIS	OTH	MALE
C	0.72	0.98	1.14	0.86	0.84
	0.70, 0.73	0.96, 0.99	1.12, 1.16	0.83, 0.90	0.83, 0.85
I	0.83	1.04	1.20	1.08	0.84
	0.82, 0.84	1.03, 1.05	1.19, 1.21	1.06, 1.09	0.84, 0.85
V	0.94	1.08	1.64	1.14	1.00
	0.89, 0.99	1.06, 1.10	1.60, 1.68	1.09, 1.19	0.98, 1.01
E	0.91	1.35	1.29	0.69	1.00
	0.77, 1.07	1.31, 1.40	1.23, 1.36	0.59, 0.82	0.93, 1.08
T	0.90	1.10	1.31	1.40	1.07
	0.86, 0.96	1.07, 1.12	1.23, 1.39	1.33, 1.48	1.05, 1.08
S	1.07	1.02	1.19	1.12	1.04
	0.64, 1.79	0.9, 1.17	0.98, 1.45	0.96, 1.31	0.94, 1.16
OVERALL	0.90	1.13	1.31	1.13	0.90
	0.89, 0.90	1.12, 1.14	1.30, 1.32	1.12, 1.15	0.89, 0.90
**b. Positivity risk ratios & 95% confidence intervals**					
**CIVET Cohort**	**RACE** *				**SEX** **
	API	BLA	HIS	OTH	MALE
C	1.92	2.59	5.30	1.95	1.17
	1.79, 2.07	2.46, 2.74	5.02, 5.59	1.71, 2.22	1.14, 1.21
I	1.54	2.00	3.98	1.28	1.19
	1.49, 1.60	1.92, 2.09	3.88, 4.09	1.19, 1.37	1.16, 1.21
V	1.45	1.79	5.38	2.08	1.19
	1.20, 1.75	1.69, 1.89	5.13, 5.65	1.84, 2.36	1.14, 1.24
E	1.06	1.48	1.42	1.06	1.39
	0.60, 1.88	1.32, 1.65	1.20, 1.69	0.60, 1.88	1.04, 1.86
T	1.49	1.34	2.90	2.17	1.09
	1.26, 1.76	1.24, 1.44	2.56, 3.30	1.89, 2.50	1.03, 1.14
S	6.72	1.31	1.81	1.64	0.93
	1.93, 23.43	0.67, 2.57	0.8, 4.12	0.73, 3.70	0.57, 1.53
OVERALL	1.33	2.23	3.58	1.40	1.16
	1.29, 1.37	2.18, 2.28	3.51, 3.64	1.33, 1.47	1.14, 1.18
**c. Hospitalization risk ratios & 95% confidence intervals**					
**CIVET Cohort**	**RACE** *				**SEX** **
	API	BLA	HIS	OTH	MALE
C	1.35	1.41	1.21	0.88	1.36
	1.08, 1.67	1.20, 1.66	1.02, 1.43	0.57, 1.37	1.24, 1.49
I	0.88	1.23	1.34	0.86	1.34
	0.57, 1.37	1.11, 1.37	1.19, 1.51	0.79, 0.93	1.25, 1.43
V	1.03	0.91	0.91	1.51	1.19
	0.92, 1.15	0.68, 1.21	0.68, 1.21	0.85, 2.70	1.10, 1.30
E	1.26	1.11	0.76	0.76	3.13
	1.02, 1.54	0.82, 1.52	0.21, 2.68	0.21, 2.68	1.22, 7.99
T	1.41	1.86	2.43	1.59	1.25
	0.97, 2.06	1.59, 2.17	1.94, 3.05	1.17, 2.16	1.10, 1.42
OVERALL	1.02	1.36	0.86	1.18	1.30
	0.94, 1.10	1.28, 1.44	0.81, 0.90	1.04, 1.35	1.25, 1.36
**d. Death risk ratios & 95% confidence intervals**					
**CIVET Cohort**	**RACE** *				**SEX** **
	API	BLA	HIS	OTH	MALE
C	0.68	0.89	0.48	1.03	2.05
	0.42, 1.13	0.65, 1.22	0.33, 0.69	1.02, 1.04	1.62, 2.60
I	1.03	0.48	0.91	0.33	1.40
	1.02, 1.04	0.37, 0.63	0.69, 1.18	0.27, 0.40	1.19, 1.64
V	0.35	0.90	0.90	0.84	1.40
	0.25, 0.49	0.49, 1.63	0.49, 1.63	0.12, 5.98	1.13, 1.74
E	1.64	2.22	1.68	1.68	.
	1.02, 2.63	1.23, 4.01	0.25, 11.43	0.25, 11.43	.
T	1.01	1.44	1.69	1.85	2.29
	1.01, 1.02	0.83, 2.51	0.68, 4.16	0.75, 4.58	1.45, 3.62
OVERALL	0.51	1.17	0.41	1.12	1.70
	0.41, 0.64	1.03, 1.32	0.35, 0.47	0.84, 1.50	1.53, 1.89

*Reference Group for RACE: White

**Reference Group for SEX: Female

Abbreviations: API = Asian/Pacific Islander; BLA = Black; HIS = Hispanic; OTH = Other Race.

Patterns in overall positivity percentages (Fig 1B) followed those in testing, with the highest positivity risk among Hispanic patients (RR = 3.58 [3.51–3.64]; Table 3b). However, while more women sought testing, Hispanic men were more likely to test PCR positive than Hispanic women (17% female vs. 20% male: RR = 1.18 [1.15–1.21]). When controlling for race/ethnicity, men were more likely to test positive than women (RR = 1.16 [1.14–1.18]).

The proportions of those testing positive who were hospitalized (Fig 1C) or died (Fig 1D) were significantly different by race/ethnicity (p<0.001) and sex (p<0.001). When controlling for race/ethnicity, men were more likely to be hospitalized (RR = 1.30 [1.25–1.36]; Table 3c) and to die (RR = 1.70 [1.53–1.89]; Table 3d). Diverging from the racial differences in testing and positivity, hospitalization (RR = 1.36 [1.28–1.44]) and mortality (RR = 1.17 [1.03–1.32]) were highest among Black patients.

### Racial/ethnic and sex differences by health system type

**Fig 2:**
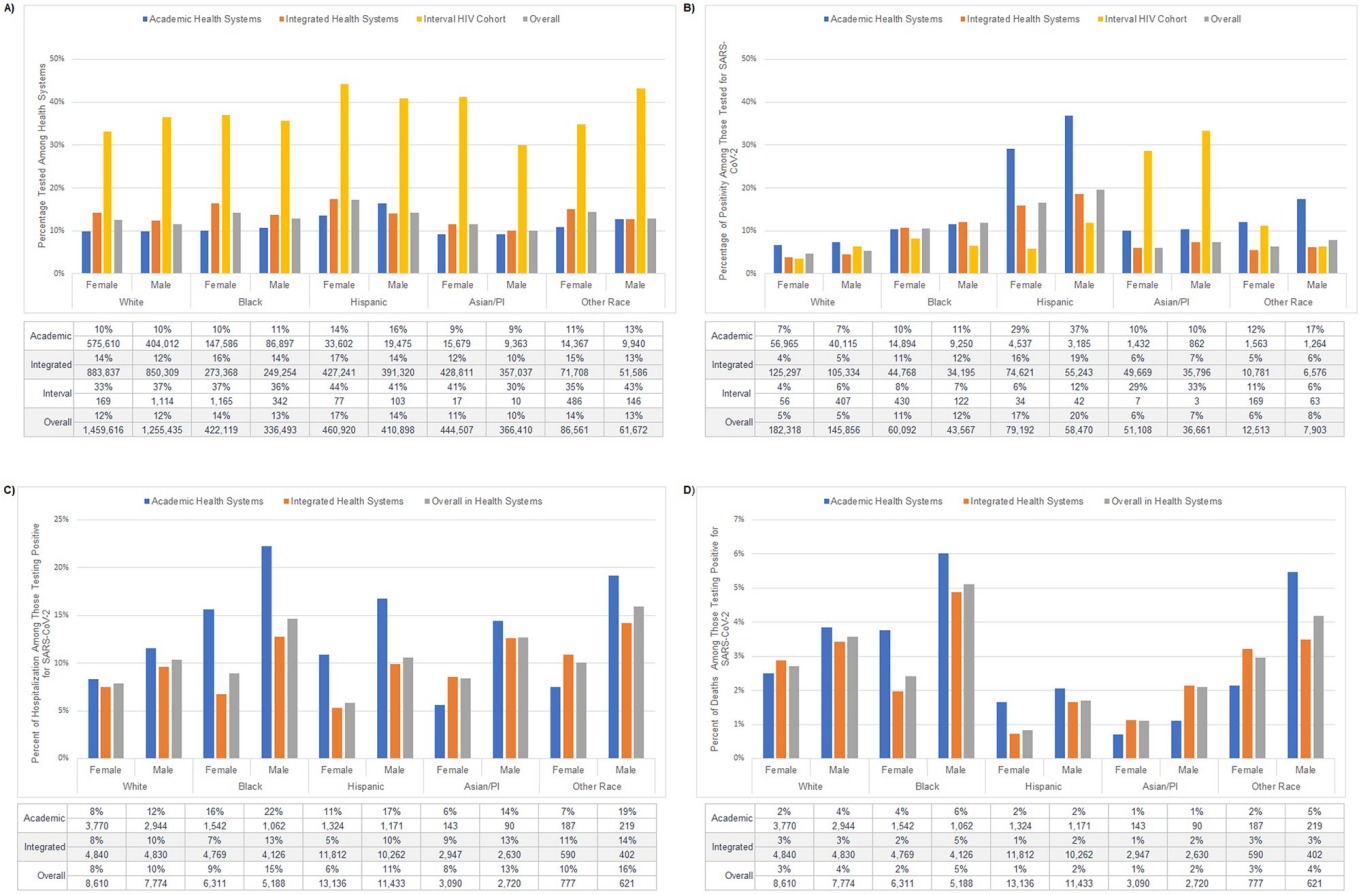
Racial/ethnic and sex differences in COVID-19 outcomes in clinical cohorts stratified by health system classification for (A) SARS-CoV-2 testing, (B) positivity, (C) hospitalization, and (D) death. Overall percentages are calculated among clinical cohort population (N = 5,304,631). Asian/PI = Asian/Pacific Islander.

The racial/ethnic differences in the proportion tested were attenuated in integrated health systems, but not negated. When stratified by health system classification, testing percentages between the integrated health systems and academic health systems varied (Fig 2A). Testing rates were higher across all racial/ethnic groups stratified by sex in integrated health systems compared with academic systems, except for Hispanic males (14% vs. 16%; RR = 0.86 [0.84–0.89]).

Positivity varied by health system type (Fig 2B). Notably, the percent of Hispanic patients who tested positive was much higher in the academic health systems than the integrated health systems, largely driving the high positivity percentages among Hispanic patients overall.

When stratified by health system, the percent of patients hospitalized for COVID-19 (Fig 2C) was lower in integrated health systems (RR = 0.63 [0.60–0.66]) when controlling for race and sex. Mortality rates were also lower in the integrated health systems (RR = 0.83 [0.74–0.94]). Deaths were higher among Black patients in academic health systems as compared to integrated health systems (RR = 1.39 [1.13–1.71]; Fig 2D).

Within the interval cohort, testing proportions (Fig 2A) were highest among Hispanic patients (42%) compared to all other racial/ethnic groups (36–37%, controlling for sex). When examining the interaction between race/ethnicity and sex, the interval cohort saw the highest testing percentages among males identifying as “other” race (43%) and Hispanic females (44%), and lowest among API males (30%) and White females (33%). Among those tested, positivity (Fig 2B) was significantly higher among API patients compared to all other racial/ethnic groups (30% vs. 6–10%); this held true after examining the interaction of race/ethnicity and sex. Hospitalization and death data were not included for the interval cohort as these data were self-reported and not confirmed.

## Discussion

The COVID-19 pandemic has brought the issues of health disparities and racial inequities in the United States to the forefront. This CIVET cohort analysis bolsters prior smaller, regional, and single-center studies [8, 21] by finding similar racial disparities in a large national cohort collaboration. Hispanic patients in the CIVET cohort had the highest proportion of positivity, [10, 21, 22, 35] while more severe COVID-19-related outcomes (hospitalization and death) were highest among Black patients [7, 22]. The size of the CIVET cohort allowed us to evaluate rates of COVID-19-related testing, positivity, hospitalization, and mortality stratified by race/ethnicity as well as sex. This analysis found the possibility of an interaction between race/ethnicity and sex, such that Hispanic and Black men were at particular risk for testing positive and adverse outcomes, respectively. We report here on racial and sex differences across different care delivery models, demonstrating that the underlying susceptibility to infection and progression to more severe outcomes are not attributable to care delivery systems.

The observed racial differences have been previously associated with systemic inequities among vulnerable populations. Multiple factors may contribute to the higher risk of infection with SARS-CoV-2 and severe complications. Type of work and inability to social distance (e.g., essential workers, public-facing occupations) increased exposure to SARS-CoV-2 for many in the US, particularly minority workers [36]. Lower socioeconomic status and lack of access to healthcare are linked to a higher prevalence of chronic diseases among Black and Hispanic populations [3, 7, 10]. Additional cultural considerations, such as multi-generational housing and distrust of the medical community [37], contribute to increased infections and poorer outcomes [38]. These factors may increase the time between symptom onset and getting tested, leading to increased hospitalization and mortality. How these factors work alone and in synergy with each other need to be evaluated to understand why Black and Hispanic Americans had a higher burden of disease. While this cohort did not have economic or housing status data, we did have site-level data to describe access to care.

We have shown significant differences in COVID-19 testing and outcomes by health system type. The interval cohort includes participants who are closely affiliated with their healthcare providers, many having participated in the HIV study for decades. This group had a high rate of testing, which indicates they had the access and knowledge necessary to secure testing. Similarly, patients of the integrated health systems are established members of the system and have a well-defined entry point for all care, facilitating testing access. In contrast, the lowest levels of testing were seen in the academic setting, where patients may need to secure testing independently or are using the health system for the first time. Confirmation of infection is key to slowing the spread of disease, and while COVID-19 testing was in most places without patient cost, this evolved over time and early access to testing was more limited [5, 39]. Though many factors may influence this, we argue that different health systems afforded different opportunities for testing.

When comparing integrated health systems, with lower perceived barriers to care, to academic systems, we found that racial disparities were reduced but persisted in the integrated healthcare setting. While access to care is important, it represents the end of the disparity pathway, not the beginning; individual, household, and structural differences among Black and Hispanic populations and men may be driving forces leading to disparities in testing and outcomes related to COVID-19 regardless of access. Though we could not measure these factors directly, our results are consistent with disparities seen throughout the US. These results highlight the overarching need to address racial disparities for the long-term, not just in response to this pandemic. These findings should bolster the evidence to inform culturally competent policy changes to address health inequalities [38].

In addition to describing differences in COVID-19 testing and adverse outcomes, our findings correspond to the next phases of the pandemic: vaccination and long-term health consequences of COVID-19. The swift development and deployment of multiple vaccines have led to declines in cases, adverse outcomes, and deaths nationally [40]; however, differences in vaccination rates have mirrored those seen in this and other studies [41, 42]. This may reflect an association between testing hesitancy and vaccine hesitancy, for which many of the same tactics can be employed. Furthermore, demographic trends among COVID-19 cases and severe outcomes may extend to post-acute sequelae of COVID-19, known as PASC.

### Limitations

This study does have limitations. To expedite data sharing and protect patient privacy, aggregated datasets were compiled by each cohort and submitted to KPMAS for analysis. While these summary-level data limited our capabilities with the analytic approach and did not allow for deeper dives into patient-level effects, the large sample size and diversity within each cohort provided adequate data to describe differences by race/ethnicity and sex in COVID-19 outcomes over an extended period. Additionally, some COVID-19-related data may have been missing due to lack of coding early in the pandemic or care that occurred outside of the represented cohorts not captured. Importantly, guidelines for testing differed by state and healthcare system over the course of the pandemic, and testing was ultimately at the discretion of clinicians and by patients to a limited extent. The known delays of death certificate data, which limited our ability to directly attribute deaths to COVID-19, may have contributed to larger variances in COVID-19 death estimations. However, our large sample size provided sufficient power for robust statistical analysis to generate findings. Further, we included only deaths among SARS-CoV-2 positive patients, if they occurred during our study period, therefore capturing inpatient deaths rather completely. We also acknowledge that no formal analysis was conducted to see if the CIVET cohorts were representative of the population; however, risk analysis attenuates this limitation by controlling for base population rates, thus maintaining the generalizability of our results.

There were limitations specific to each cohort. The interval cohort and one of the integrated health system cohorts are HIV cohort studies of persons with and without HIV, differing from the other health systems cohorts which represent whole populations; however, the proportion SARS-CoV-2 positive in the HIV cohort for this integrated health system are consistent with a national study [19]. For the interval cohort, test result and hospitalization data were self-reported (with confirmation in a subset) and subject to potential misclassification. This cohort did not have vital status post-COVID assessment available in time for this analysis so was not included in the analysis of death outcomes. Additionally, a cohort among the integrated health systems was unable to include pending lab results from SARS-CoV-2 tests. Finally, two cohorts (interval cohort and one among the integrated health systems) lacked insurance data. However, the strengths of the CIVET cohort (i.e., the heterogeneity with respect to geography, healthcare system type, number of patients, etc.) far outweigh its limitations.

## Conclusions

This study identified significant racial/ethnic differences in COVID-19 outcomes within a large diverse cohort, which were attenuated in settings with lower barriers to care (i.e., integrated health systems), but not negated. Further research is needed to understand the underlying mechanisms behind why Hispanic and Black men in America have a higher burden of disease during the COVID-19 pandemic and how to combat those disparities.

## Supporting information

S1 Table Cohort inclusion criteria. *The Kaiser Permanente Mid-Atlantic and Northern California HIV Registries are databases of members diagnosed with HIV since 1998. Primary sources used to identify HIV patients are HIV-specific laboratory tests, diagnosis by infectious disease physicians, hospital-based HIV diagnosis, and antiretroviral therapy. Abbreviations: Multicenter AIDS Cohort Study = MACS; PWH = Persons with HIV; PWoH = Persons without HIV; Women’s Interagency HIV Study = WIHS.
(PDF)
