# Supplementary material for: Differences in COVID-19 testing and adverse outcomes by race, ethnicity, sex, and health system setting in a large diverse US cohort
Source: PLoS One. 2022 Nov 23;17(11):e0276742. doi: 10.1371/journal.pone.0276742 (PMC9683575; doi:10.1371/journal.pone.0276742)
Supplement: S1 Table — *The Kaiser Permanente Mid-Atlantic and Northern California HIV Registries are databases of members diagnosed with HIV since 1998. Primary sources used to identify HIV patients are HIV-specific laboratory tests, diagnosis by infectious disease physicians, hospital-based HIV diagnosis, and antiretroviral therapy. Abbreviations: Multicenter AIDS Cohort Study = MACS; PWH = Persons with HIV; PWoH = Persons without HIV; Women’s Interagency HIV Study = WIHS. (PDF) [file pone.0276742.s001.pdf]

SUPPLEMENTAL TABLES/FIGURES

S1 Table. Cohort inclusion criteria

Cohort	Description	"In care" or "in cohort"	PWH
Kaiser Permanent Mid-Atlantic States (KPMAS)	Integrated Health system	In Care – KPMAS membership for ≥1 month in 2020	HIV registry*
Kaiser Permanente Northern California (KPNC)	Integrated Health system	In Care – KPNC membership for ≥1 month between 3/1/20 and 12/31/20	HIV registry*
MACS/WIHS Combined Cohort Study (MWCCS)	Classical interval HIV cohort study	Alive in 2020	HIV tested positive
University of North Carolina Chapel Hill (UCHCC)	Medical center cohort	≥1 encounter with UCHCC Health in 2019 and alive as of 03/01/2020	HIV diagnosis (by ICD diagnosis code)
Veterans Aging Cohort Study (VACS)	National cohort of all PWH and 1:2 demographically matched PWoH in care in the Veterans Health Administration (VA) system	Enrolled in VACS from 1996-2017 and alive in 2020	HIV diagnosis (presence of 1 inpatient or 2 outpatient ICD codes for HIV)
Vanderbilt University Medical Center (VBCCC)	Medical center cohort	Encounter with VUMC in 2019 and alive in 2020	HIV diagnosis code or problem list mention
**Purpose**: The goals of the collaboration were to: 1) leverage the long-standing partnerships of the NA-ACCORD to answer questions pertinent to the COVID-19 pandemic in the United States; 2) identify selection biases in identifying individuals with COVID-19 through testing, diagnoses, or symptoms (due to changing barriers to testing and care); and 3) share challenges and barriers in accessing COVID-19 data in different electronic health record (EHR) systems by researchers experienced with EHR-based longitudinal research.			

*The Kaiser Permanente Mid-Atlantic and Northern California HIV Registries are databases of members diagnosed with HIV since 1998. Primary sources used to identify HIV patients are HIV-specific laboratory tests, diagnosis by infectious disease physicians, hospital-based HIV diagnosis, and antiretroviral therapy.

Abbreviations: Multicenter AIDS Cohort Study = MACS; PWH= Persons with HIV; PWoH= Persons without HIV; Women's Interagency HIV Study = WIHS
